# A New Dibenz[*b*, *e*]oxepine Derivative, 1-Hydroxy-10-methoxy-dibenz[*b*, *e*]oxepin-6,11-dione, from a Marine-Derived Fungus, *Beauveria bassiana *TPU942

**DOI:** 10.3390/md10122691

**Published:** 2012-11-27

**Authors:** Hiroyuki Yamazaki, Henki Rotinsulu, Tsuyoshi Kaneko, Kazuki Murakami, Hiromu Fujiwara, Kazuyo Ukai, Michio Namikoshi

**Affiliations:** 1 Department of Natural Product Chemistry, Tohoku Pharmaceutical University, Sendai 981-8558, Japan; Email: yamazaki@tohoku-pharm.ac.jp (H.Y.); rhenki@yahoo.com (H.R.); 20711216@is.tohoku-pharm.ac.jp (T.K.); 20711445@is.tohoku-pharm.ac.jp (K.M.); 20711640@is.tohoku-pharm.ac.jp (H.F.); ukai_k@tohoku-pharm.ac.jp (K.U.); 2 Faculty of Agriculture, Indonesia Development University, Manado 95361, Indonesia

**Keywords:** dibenzooxepine derivative, 1-hydroxy-10-methoxy-dibenz[*b*, *e*]oxepin-6,11-dione, marine-derived fungus, fungal metabolites

## Abstract

1-Hydroxy-10-methoxy-dibenz[*b*, *e*]oxepin-6,11-dione (**1**) was obtained from the culture broth of a marine-derived fungus, *Beauveria bassiana *TPU942, isolated from a marine sponge collected at Iriomote Island in Okinawa, together with two known compounds, chrysazin (**2**) and globosuxanthone A (**3**). The structure of **1** was elucidated on the basis of its spectroscopic data (HREIMS, 1D and 2D NMR experiments including ^1^H–^1^H COSY, HMQC and HMBC spectra). Dibenz[*b*, *e*]oxepines are rare in nature, and only six natural products have been reported. Therefore, compound **1** is the seventh natural product in this class. Compounds **2** and **3 **showed an antifungal activity against *Candida albicans*, and **3** inhibited the cell growth against two human cancer cell lines, HCT-15 (colon) and Jurkat (T-cell lymphoma). Compound **1** did not show an apparent activity in the same bioassays.

## 1. Introduction

Microorganisms from marine environments, especially marine-derived fungus, have proven to be an attractive source of new bioactive secondary metabolites [1,2,3,4,5]. Many of the metabolites possess unique chemical structures and interesting biological activities, such as cytotoxicity against cancer cell lines, antimicrobial activity, inhibition of several enzyme activities and so on [1,2,3,4,5].

**Figure 1:**
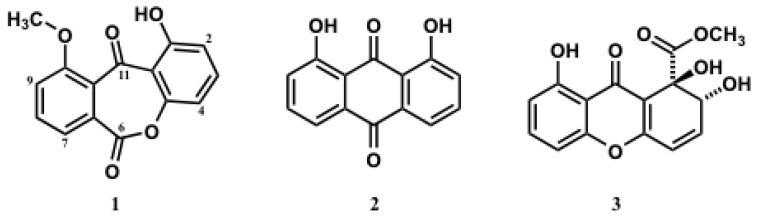
Structures of compounds **1**–**3** isolated from *Beauveria bassiana* TPU942.

In the course of our studies on bioactive components produced by marine-derived fungi isolated from tropical and sub-tropical coral reefs, we found that the EtOAc extract of the culture broth from a marine-derived fungus, *Beauveria bassiana* TPU942, isolated from a marine sponge collected at Iriomote Island, Okinawa Prefecture, Japan, showed cytotoxicity against a human T-cell lymphoma Jurkat cells. Bioassay-guided separation from the EtOAc extract led to the isolation of a new dibenz[*b*, *e*]oxepine derivative (**1**) and two known anthraquinone and xanthone derivatives, chrysazin (**2**) and globosuxanthone A (**3**). The structure of compound **1** was elucidated as 1-hydroxy-10-methoxy-dibenz[*b*, *e*]oxepin-6,11-dione (Figure 1) by the analysis of its spectroscopic data. Natural products possessing a dibenz[*b*, *e*]oxepine structure are quite rare, and only six compounds have been reported as natural products in scientific journals [6,7,8,9]. Antifungal and cytotoxic activities were exhibited by two known compounds (**2** and **3**).

We describe herein the isolation and structure elucidation of compound **1** and biological activities of isolated compounds.

## 2. Results and Discussion

The producing fungus, *B. bassiana* TPU942, was isolated from a piece of an unidentified marine sponge collected at Iriomote Island and cultured as described in Experimental Section. The culture broth and the EtOAc extract of strain TPU942 showed cytotoxicity in the screening bioassay against Jurkat cells and was separated into seven fractions (Fr. 1–Fr. 7) using a silica gel column. Compounds **1** and **2 **were isolated from Fr. 1 (eluted with 100% CHCl_3_) and **3** from Fr. 2 (CHCl_3_–MeOH = 100:1) by HPLC (ODS).

Compound **2** was identified as chrysazin [10], a ubiquitous anthraquinone derivative isolated from several organisms, from the spectroscopic data. The structure of compound **3** was assigned on the basis of its spectral data and comparison of these data with those of the reported values for globosuxanthone A [11]. Globosuxanthone A (**3**) was originally isolated from *Chaetominum globosum*, and a cytotoxicity against seven human solid tumor cell lines, accumulation of cells at either G2/M or S phase and induction of apoptosis have been described [11].

Compound **1** showed a molecular ion peak at *m/z* 270 [M]^+^ in the EIMS, and the molecular formula C_15_H_10_O_5_, 11 degrees of unsaturation, was determined from HREIMS [*m/z* 270.0531 [M]^+^, Δ +0.3 mmu] and NMR data. IR absorptions of **1 **at 1735 and 3435 cm^−1^ suggested the presence of carbonyl and hydroxyl groups in the molecule. The ^13^C NMR spectrum showed 15 resolved signals, which were classified into one oxygenated methyl, six sp^2^ methine, three sp^2^ quaternary, three oxygenated sp^2^ quaternary and two carbonyl carbons by the analysis of 1D and 2D NMR spectra (Table 1). The ^1^H NMR spectrum displayed 10 proton signals, and two signals at δ 4.03 and 12.2 were assigned as a hydroxy proton (1-OH) and methoxy protons (10-OMe), respectively (Table 1). The connectivity of carbons and protons was established by the HMQC correlations.

**Table 1:** ^13^C (100 MHz) and ^1^H NMR (400 MHz) data for compound **1** (CDCl_3_).

No.	δ_C_	δ_H _(*J* in Hz)	HMBC
1	161.8	-	1, 2, 11a
2	111.0	6.82 dd (8.3, 1.0)	1, 4
3	137.1	7.61 t (8.5)	1, 4a
4	106.9	6.94 dd (8.3, 1.0)	2, 4a
4a	155.8	-	-
5			
6	156.1	-	-
6a	133.7	-	-
7	119.5	7.56 dd (8.3, 1.0)	6, 9, 10a
8	135.0	7.77 dd (8.5, 7.1)	6, 6a
9	122.7	7.33 dd (7.3, 1.0)	10, 10a
10	169.5	-	-
10a	117.5	-	-
11	181.0	-	-
11a	109.0	-	-
1-OH	-	12.2 s	1, 2, 11a
10-OCH_3_	53.1	4.03 s	10

**Figure 2:**
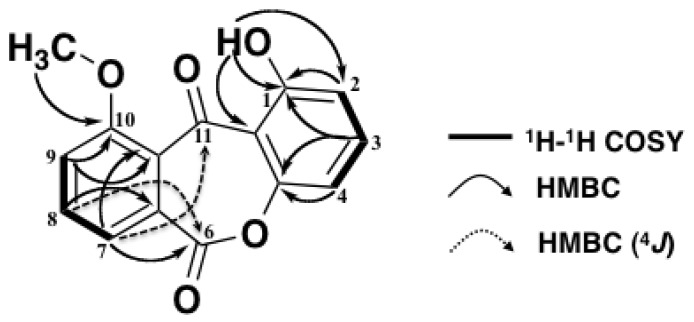
^1^H-^1^H COSY and HMBC correlations of compound **1**.

The presence of two 1,2,3-trisubstituted aromatic rings was assigned by the ^1^H–^1^H COSY and HMBC correlations of the ^1^H NMR signals at δ 6.82 (H-2), 7.61 (H-3), 6.94 (H-4), 7.56 (H-7), 7.77 (H-8) and 7.33 (H-9) (Figure 2). HMBC correlations were observed from 1-OH (δ 12.2) to C-1 (δ 161.8), C-2 (δ 111.0) and C-11a (δ 109.0) and from 10-OMe (δ 4.03) to C-10 (δ 169.5). Therefore, an OH group was attached at the C-1 position and an OMe group at C-10. An HMBC correlation from H-7 (δ 7.56) to C-6 (δ 156.1) and from H-8 to C-6 (^4^*J*), and the ^13^C chemical shift of C-4a (δ 155.8), an oxygenated sp^2^ carbon, revealed that C-4a and C-6a were connected via C-6 by an ester bond. Therefore, one remaining carbonyl group (δ 181.0, C-11) was inevitably attached between C-10a and C-11a [6,7,8,9]. This assignment was supported by an HMBC correlation from H-7 to C-11 (^4^*J*). Thus, the structure of compound **1** was assigned as 1-hydroxy-10-methoxy-dibenz[*b*, *e*]oxepin-6,11-dione.

**Figure 3:**
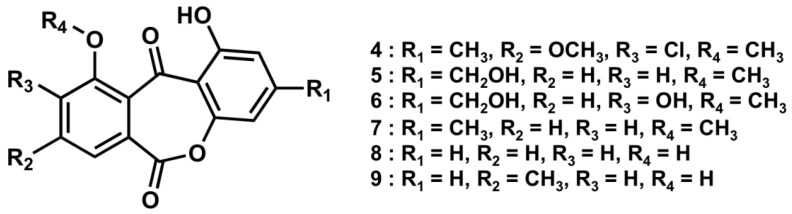
Structures of related compounds **4**–**9**.

Dibenz[*b*, *e*]oxepines are rare metabolites, and, thus far, only six natural products (**4**–**9**, Figure 3) have been reported [6,7,8,9]. Compound **1** is, therefore, the seventh example of this class of natural products. ^13^C and ^1^H NMR data for **1** were very similar to those of the reported values for **5** [7].

Compounds **1**–**3** were tested for their antimicrobial activity against four test microorganisms (fungus *Mucor hiemalis*, yeast *Candida albicans*, Gram-positive bacterium *Staphylococcus aureus* and Gram-negative bacterium *Escherichia coli*) using the paper disk method and for their cytotoxicity against two human cancer cell lines (colon adenocarcinoma HCT-15 and T-cell leukemia Jurkat cells) using the MTT method [12] (Table 2). Compounds **2** and **3** showed inhibition zone of 7 mm against *C*. *albicans* at the concentration of 10 μg/disk. Compound **3** inhibited the cell proliferation against HCT-15 and Jurkat cells with IC_50_ values of 10.7 and 2.3 μM, respectively. On the other hand, compound **1** did not show apparent activity in these bioassays. Further study on biological activity of compound **1** is now in progress.

**Table 2:** Biological activities of compound **1**–**3**.

Compound	IC_50_ (μM)								Inhibition zone (mm)											
	Cytotoxicity								Antimicrobial activity (10 μg/disk)											
	HCT-15				Jurkat				*S. aureus*			*E. coli*			*C. albicans*			*M. hiemalis*		
**1**	>	30			>	30				– ^a^			– ^a^			– ^a^			– ^a^	
**2**	>	30			>	30				– ^a^			– ^a^			7			– ^a^	
**3**		10.7				2.3				– ^a^			– ^a^			7			– ^a^	

^a^ Not active.

## 3. Experimental Section

### 3.1. General

EI mass spectra were obtained by a JEOL JMS-MS 700 mass spectrometer (Tokyo, Japan). ^1^H and ^13^C NMR spectra were recorded on a JEOL JNM-AL-400 NMR spectrometer (400 MHz for ^1^H and 100 MHz for ^13^C) in DMSO-*d*_6_ (δ_H_ 2.49, δ_C_ 39.5) or CDCl_3_ (δ_H_ 7.26, δ_C_ 77.0). UV spectra were measured on a Hitach U-3310 UV-Visible spectrophotometer (Tokyo, Japan) and IR spectra on a PerkinElmer Spectrum One Fourier transform infrared spectrometer (Waltham, MA, USA). Preparative HPLC was carried out with a Hitachi L-6200 system.

### 3.2. Materials

Fetal bovine serum (FBS) and other culture materials were purchased from Invitrogen (Carlsbad, CA, USA). 3-(4,5-Dimethylthiazol-2-yl)-2,5-diphenyltetrazolium bromide (MTT) was purchased from Sigma-Aldrich (St. Louis, MO, USA). All other chemicals and organic solvents were purchased from Wako Pure Chemical Industries Ltd. (Osaka, Japan).

### 3.3. Fermentation and Isolation

Three pieces of an unidentified marine sponge collected in the coral reef of Iriomote Island in Okinawa, Japan were incubated on a 1/2 PDA plate (Difco Laboratories, Detroit, MI, USA). The strain TPU942 was grown from the sponge body and inoculated into a slant (1/10 YSA). The fungus was identified as *Beauveria bassiana* by the comparison of 217 bp ITS1 rDNA sequence (100% match).

A slant culture of strain TPU942 grown on 1/10 YSA (0.020% yeast extract, 0.10% soluble starch, and 1.5% agar; dissolved in 90% sea water and adjusted to pH 6.0 before sterilization) was inoculated into a 500-mL Erlenmeyer flask containing 100 mL of the seed medium (2.0% glucose, 0.50% polypeptone, 0.050% MgSO_4_·7H_2_O, 0.20% yeast extract, 0.10% KH_2_PO_4_ and 0.10% agar; adjusted to pH 6.0 before sterilization). The flask was shaken reciprocally for three days at 27 °C to obtain the seed culture, which was then transferred to the production medium (3.0% sucrose, 3.0% soluble starch, 1.0% malt extract, 0.30% Ebios (Asahi Food & Healthcare Co. Ltd., Tokyo, Japan), 0.50% KH_2_PO_4_ and 0.050% MgSO_4_·7H_2_O; adjusted to pH 6.0 before sterilization). The production culture was carried out at 27 °C for seven days under the agitation condition. The seven-day-old whole broth (2.0 L) was extracted with 2.0 L of acetone. The extract was filtered and concentrated to remove acetone, and the aqueous solution was extracted with ethyl acetate. The EtOAc extract was dried over Na_2_SO_4_ and concentrated *in vacuo* to dryness to yield a red brown material (1088.3 mg), and the residue was suspended in CHCl_3_ and adsorbed on a silica gel column (100 g). The silica gel column was eluted stepwise with each 500 mL of CHCl_3_, a mixture (v/v) of CHCl_3_–CH_3_OH (10:1, 5:1 and 1:1) and CH_3_OH into seven fractions (Fr. 1–Fr. 7). An active Fr. 1 (CHCl_3_ eluate) was concentrated *in vacuo* to dryness to give a brown oil (112.9 mg). A portion (40 mg) of Fr. 1 was purified by a preparative HPLC [column; PEGASIL ODS (Senshu Scientific. Co. Ltd. Tokyo, Japan), 10 × 250 mm; solvent, 90% CH_3_OH; detection, UV at 254 nm; flow rate, 2.0 mL/min] to give compounds **1** (eluted at 11.0 min) and **2** (eluted at 17.8 min) as a pale yellow solid (3.0 mg) and an orange solid (24.1 mg), respectively. The second active Fr. 2 (CHCl_3_-CH_3_OH = 100:1) was concentrated to yield a brown oil (368.9 mg), and 50 mg of residue was purified by a preparative HPLC (same conditions as Fr. 1) to yield compound **3** (eluted at 7.6 min) as a white solid (4.7 mg).

**1-Hydroxy-10-methoxy-dibenz[*b*, *e*]oxepin-6,11-dione (1)**: obtained as a pale yellow solid; UV λ_max_ (MeOH) nm (ε): 230 (39200), 254 (36900), 282 (10800), 300 (10200), 363 (3600); IR ν_max_ (KBr) cm^−1^: 3435, 1735, 1650, 1604, 1490 1290; HREIMS (*m/z*) found: 270.0531, calcd: 270.0528 [M]^+^ for C_15_H_10_O_5_; ^1^H and ^13^C NMR data, see Table 1.

**Chrysazin (2)**: obtained as an orange solid; EIMS (*m/z*): 240 [M]^+^; ^1^H NMR (CDCl_3_) δ 7.26 (2H, dd, *J* = 8.5, 1.2 Hz), 7.65 (2H, t, *J = * 8.0 Hz), 7.78 (2H, dd, *J = * 7.5, 1.2 Hz), 12.0 (2H, s, 2 × OH); ^13^C NMR (CDCl_3_) δ 115.8, 120.0, 124.6, 133.5, 137.2, 162.5, 181.5, 193.0.

**Globosuxanthone A (3)**: obtained as a white solid; EIMS (*m/z*): 304 [M]^+^; ^1^H NMR (DMSO-*d*_6_) δ 4.28 (1H, d, *J* = 4.4 Hz), 6.49 (1H, d, *J* = 10.1 Hz), 6.61 (1H, dd, *J* = 9.9, 4.1 Hz), 6.82 (1H, d, *J* = 8.2 Hz), 7.07 (1H, d, *J* = 8.7 Hz), 7.66 (1H, t, *J* = 8.2 Hz), 12.4 (1H, s, OH); ^13^C NMR (DMSO-*d*_6_) δ 51.7, 71.4, 75.0, 107.3, 110.1, 111.2, 114.6, 119.6, 136.0, 141.3, 154.8, 159.7, 159.9, 171.6, 180.6.

### 3.4. Antimicrobial Assay

The growth inhibitory activity was examined by the paper disk method against *Mucor hiemalis* IAM 6088 (fungus), *Candida albicans* IFM 4954 (yeast), *Staphylococcus aureus* IAM 12544T (Gram-positive bacterium) and *Escherichia coli* IAM 12119T (Gram-negative bacterium) as test microorganisms.

### 3.5. Cytotoxicity Assay

HCT-15 and Jurkat cells were obtained from the Center for Biomedical Research, Institute of Development, Aging and Cancer, Tohoku University (Miyagi, Japan). The cell lines were cultured in RPMI-1640 medium. The medium was supplemented with 10% fetal bovine serum, 100 units/mL penicillin, and 100 μg/mL streptomycin. Exponentially growing cells, cultured in a humidified chamber at 37 °C containing 5.0% CO_2_, were used for experiments.

Cytotoxic activity was evaluated using the colorimetric MTT assay [12]. HCT-15 (1.0 × 10^4^ cells in 100 μL) or Jurkat cells (2.0 × 10^4^ cells in 100 μL) were added to each well of a 96-well plastic plate (Corning Inc., Corning, NY, USA). A sample (1.0 μL in CH_3_OH) was added to each well to make the final concentration from 0 to 30 μM, and the cells were incubated for 48 hr at 37 °C. MTT (10 μL of 5.5 mg/mL stock solution), and a cell lysate solution (90 μL, 40% *N*, *N*-dimethylformamide, 20% sodium dodecyl sulfate, 2.0% CH_3_COOH and 0.030% HCl) were added to each well and the plate was shaken thoroughly by agitation at room temperature overnight. The optical density of each well was measured at 570 nm using an MTP-500 microplate reader (Corona Electric Co., LTD., Ibaraki, Japan).

## 4. Conclusions

A new 1-hydroxy-10-methoxy-dibenz[*b*, *e*]oxepine-6,11-dione (**1**) and two known compounds, chrysazin (**2**) and globosuxanthone A (**3**), were obtained from a marine-derived fungus, *B. bassiana*, strain TPU942, isolated from a marine sponge collected in Iriomote Island, Okinawa. Bioactivities observed by the extract of culture broth were reproduced by two known compounds, but compound **1** was not active in the same bioassays. Compound **1** had a rare dibenz[*b*, *e*]oxepine structure, and only six natural products have thus far been reported.
